# Grand Adventure of Augmented Reality in Landscape of Surgery

**DOI:** 10.29252/wjps.8.2.135

**Published:** 2019-05

**Authors:** Fatemeh Salehahmadi, F Hajialiasgari

**Affiliations:** 1 Computer Engineering Department, Islamic Azad University, Bushehr Branch, Dashtestan Campus, Borazjan Motaghin Research Center, Bushehr, Iran;; 2 Information Technology Department, Electronic Health Group, Tehran University of Medical Sciences, Tehran, Iran

**Keywords:** Augmented reality, Surgery, Rewards, Drawbacks

## Abstract

Computer as an integral part of continual advancements in medicine has experienced tremendous development to minimize the risks and improving the precision of the surgery. Our review included multi-disciplinary publications in English from 2014 to 2017 using Springer, Oxford library, Elsevier, PubMed, Google Scholar, and Springer search engines using terms of “augmented reality (AR), “plastic surgery,” and “surgery “ and “Augmented Reality Ethics and challenges”. It was shown that AR has been successfully effective in different branches of surgery, but with concerns and challenges like acceptance, privacy, different physical, security and behavioral threats. To come over them partially, a methodological approach for cyber threat landscape proactive exploration has been suggested.

## INTRODUCTION

The Continuance of Alvin Tofler‘s third wave, electronic age, which began after 2010, is the current virtual world with extremity enhancement in information technology (IT) , 3-D spaces as well as knowledge-based environment.^1^ The integration of information technology in the 21^st^ century in the medical sector offers the evolution in information-focused healthcare, which can potentially lead to a revolution in patient treatment. Surgeons are regularly looking for technologies that would enhance their operating environment and to offer a better surgical and patient experience.^1^

Examples of such innovations include fiber optics allowing the advent of minimal access and robotic surgery leading to developments of systems such as the da Vinci robot over a decade ago. These tools have historically come at a considerable cost, but over the last decade became cheaper and increasingly available. New computational paradigms are emerging with rapid advancement and miniaturization of real-time visualization platforms. Smartphones are now commonplace with micro-processing powers rivalling desktop computers. The near ubiquitous use of smartphones by doctors has driven an increasing use of technology in health care.^2^

Medical applications and instant access to web-based resources now guide clinical practice. This has allowed for the development of powerful wearable technologies that can provide high fidelity audio-visual data to the surgeon whilst operating. The recent emerging role and integration of augmented reality (AR) in healthcare is ripe for translation into this data rich field and warrants consideration for future applications to enhance the surgical experience. This viewpoint aims to review the applications, limitations and legal pitfalls of these devices across surgical specialties and imagines what the future surgical landscape may reveal.^2^

On the other hand, the whole history of humanity is an enormous accumulation of data. Information has been stored for thousands of years and data has become an integral part of history, politics, science, economics and business structures, and now even social lives. This trend is clearly visible in social networks such as Facebook, Twitter and Instagram, where users produce an enormous stream of different types of information daily (music, pictures, text, etc.). As data processing rates are growing continuously, a situation may appear when traditional analytical methods would not be able to stay up to date, especially with the growing amount of constantly updated data, which ultimately opens the way for Big Data technologies.^3^

The current activity in the field of Big Data visualization is focused on the invention of tools that allow a person to produce quick and effective results working with large amounts of data. One of the more promising methods for improving upon current Big Data visualization techniques is its correlation with AR that is suitable for the limited perception capabilities of humans.^3^

Augmented reality (AR)

Have you ever played” Pokémon game?” or have you happened to use “Erase Brusher”, “Rewind videos”, “Type “hashtag#” or “Add effects” in Instagram? All are AR capabilities. AR (to augment, lat. augmentare: to improve, to supplement, to enhance) is the enrichment of the perceived reality by means of artiﬁcial virtual content. AR denotes a technique to combine a real world and virtual objects which are artificially generated digital content by a computer. AR can allow the user to see 3D virtual objects superimposed upon the real world. With the help of AR in medicine, a surgeon can see hidden organs inside a body and improve the perception of treatment procedure by interacting with the real world.^4^

AR components

A complete AR system requires at least three components including a tracking component, a registration component, and a visualization component. A fourth component, a spatial model (i.e., a database) stores information about the real world and about the virtual world. The real-world model is required to serve as a reference for the tracking component, which must determine the user’s location in the real world. The virtual-world model consists of the content used for the augmentation. Both parts of the spatial model must be registered in the same coordinate system. AR uses a feedback loop between human user and computer system. The user observes the AR display and controls the viewpoint. The system tracks the user’s viewpoint, registers the pose in the real world with the virtual content, and presents situated visualizations.^5^

How does AR work?

AR combines real and digital elements in one of three ways including using a Head-Mounted Display (HMD), placing the visual information close in front of the user’s point of view; using handheld devices, most commonly smartphones and tablets; and computer-generated overlay that is placed directly on real objects using projects or devices known as Spatial Displays. AR has been used to make inroads in the medical domain. For example, AR has been used to give surgeons information about the position of internal organs and the adjustments needed for needle biopsy.^6^^,^^7^

AR Goals and Motivations

Augmented reality is used in many fields including medicine, education, manufacturing, and entertainment. Hardware components for augmented reality are processor, display, sensors and input devices. Modern mobile computing devices like smartphones and tablet computers contain these elements which often include a camera and MEMS sensors; such as accelerometer, GPS, and solid state compass, making them suitable AR platforms.^8^

The goals of the AR are (1) to make the life of the user easier through providing the virtual information to his adjacent environment as well as to any indirect view of the real-world environment like the live-video stream; (2) to develop the user’s insight into and communications with the real world. The virtual reality or the virtual environment as named by Milgram engages users totally in an artificial world without seeing the real one; (3) To boost the sense of reality through laying virtual items over the real world in real time. AR not only adds items in real word but also represents useful digital information in real world;^8^ (4) To shift through huge datasets to establish patterns and to manage bed space and medication tracking; (5) To read medical images and to integrate this information to be used in the vital services; and (6) To enhance one’s current perception of reality, whereas in contrast, virtual reality replaces the real world with a simulated one.^9^

The application of AR in healthcare is two-fold for doctors; firstly there is the training and education aspect, allowing trainee physicians to lift their heads from their textbooks and better visualize the health issues they will one day be treating; and secondly, the technology will monumentally enhance their ability to diagnose and treat conditions by allowing them to access real-time data and patient information faster than ever before.^10^ The Vein Viewer Vision system projects the patient’s vascular network onto the skin to help with needle insertion.^11^ The Speed-up Robust Feature (SURF) descriptor is applied to an endoscopic video of a liver.^12^^,^^13^

## DISCUSSION

Medicine had always relied on technology such as scalpels, probes and materia medica (the body of remedial substances used in the practice of medicine.).^14^ It is considered to be one of the most effective applications of AR. However, by the start of the 21^st^ century, new instruments were available to study, diagnose and treat the body. Today, hospitals worldwide use complex, computerized machines to image the body or assist its function called “AR”. 

Ten AR Apps for healthcare are (i) AccuVein helps providers locate patients’ veins more accurately for injections; (ii) VA-ST helps those with impaired vision to see; (iii) Brain Power works to teach life skills to children and adults on the autism spectrum; (iv) Eye Decide allows healthcare professionals to demonstrate how certain conditions impede eyesight; (v) Surgical Navigation Advanced Platform (SNAP) lets physicians demonstrate their plans for a surgery; (vi) Saagara is aimed at improving individuals’ overall physical/mental health and well-being using AR technologies; (vii) VR Dentist is a dental app that uses virtual and augmented reality for educational purposes; (viii) smARtsKin (in development) superimposes relevant patient to contour onto real-time camera feeds of patients undergoing radiation therapy; (ix) Anatomy 4D visualizes detailed bone structures and organ systems when the device is pointed at special downloaded templates; and (x) Google Glass, despite being discontinued, continues to play a vital role in the development of many of the AR applications in the medical field.^15^

The traditional teaching of anatomy usually involves use of an anatomical atlas, time spent in the dissection room and fixed prosecutions. AR is being used to deliver a better appreciation of structures in virtual or real space to ease the transition from the learning to the clinical environment. An example of this is using Microsoft Kinect to produce an interactive ‘digital mirror’ of the leaner. This digital mirrored image is then augmented by anatomical datasets to visualize structures such as musculature, superimposed on the user’s own arm.^16^

Telementoring would enable juniors to share a real time view of complex cases with an off-site senior member of the team for immediate advice. Specialities whereby visual review is required would benefit from, such as injury assessment, patient monitoring and trauma management. Telementoring is a safe option for providing expert diagnosis and opinion which can reduce the likelihood of mismanagement and unnecessary patient transfer. Likewise, Multidisciplinary Team Management (MDT) requires a careful coordination of different disciplines at one site and is often fraught with difficulties in accessibility.^16^

AR has the potential to impact on surgery in a number of novel ways as discussed above, especially in the arena of surgical training in the virtual surgical environment*.* However, real-time enhancement of the surgical procedure remains a slightly tentative application. It is not yet validated that surgery can be enhanced with AR and in some instances, it could be distracting. Some features may be useful of systems like GG where with voice activation the operator could communicate beyond the theatre environment, retrieve images and test results without breaking scrub. Real-time updates regarding the progress of the trauma list would reduce unnecessary fasting of patients in the event of a delay in theatre.^16^

Real-time augmentation of surgery usually involves the blending of acquired 3D imaging with surgical reference points. Novel applications of AR include use to project optimal port placement on the abdomen for laparoscopic surgery; using AR to identify the position of sentinel nodes with 3D freehand single photon emission computed tomography; and using this with near infra-red spectroscopy to provide visual guidance in lymph node dissection in cancer surgery. Specialized near infrared (NIR) devices have been developed for the detection of tissue vascularity using indocyanine green (ICG) dye. The use of ICG in lymphatic surgery is already well developed to help identify vessels and check for their patency hence the move from microscope to HMD is a likely future development. AR technology would also be able to seamlessly project diagnostic images intra-operatively for surgical planning to guide surgeons with optimal incisions and approach.^16^

Companies are using the technology to provide patients with an augmented experience of what they can expect from surgery. Crisalix are promoting virtual aesthetics planning whereby after obtaining the patients attributes, the software can virtually demonstrate the likely changes in aesthetics such as breast enhancement. This improves documentation, communication, and education of clinicians, which will have implications for quality of service and patient safety. In addition, a case study demonstrated that using Oculus Rift (The Oculus Rift is a virtual reality headset developed and manufactured by Oculus VR, a division of Facebook Inc., released on March 28, 2016.) with distraction software reduced the level of pain experienced by a burns’ patient during occupational therapy. Other AR systems offered similar results for pain control in burns’ patients undergoing wound debridement.^16^

All of those counted features of AR, arises following question in our minds: (a) Whether or not, AR is useful in surgery? (b) How AR services can help physicians? (c) What are AR application limits? (d) How we can overcome AR limitations? To answer above questions, some search engines and articles on “AR usages in surgery and healthcare” have been chosen to study: Despite its long history, AR has only recently made its debut in medical practice, being applied primarily to navigational surgery. This involves taking data from preoperative imaging and using anatomical anchors in the operating field to link or register the two representations in real time.^17^

In a survey on “increasing intraoperative awareness to scattered radiation in interventional procedures by combining AR, Monte Carlo simulations and wireless dosimeters”, surgical staff performing image-guided minimally invasive surgical procedures are chronically exposed to harmful ionizing radiation. Currently, no means exist to intraoperatively depict the 3D shape and intensity of scattered radiation fields or to assess the body-part exposure of clinicians. A system for simulating and visualizing intraoperative scattered radiation using AR has been proposed.^17^

A multi-camera RGBD system has been used to obtain a 3D point cloud reconstruction of the current room layout. The positions of the clinicians, patient, table and C-arm are used to build a radiation propagation simulation model and compute the deposited dose distribution in the room. Examiners have been worn wireless dosimeters to calibrate the simulation and to evaluate its accuracy at each time step. The computed 3D risk map is shown in an augmented reality manner by overlaying the simulation results onto the 3D model. Results showed that several 3D visualizations showing scattered radiation propagation, clinicians’ body-part exposure and radiation risk maps under different irradiation conditions are proposed. The system is evaluated in an operating room equipped with a robotized X-ray imaging device by comparing the radiation simulation results to experimental measurements under several X-ray acquisition setups and room configurations. Then the proposed system is capable to display intraoperative scattered radiation intuitively in 3D by using AR. This can have a strong impact on improving clinicians’ awareness of their exposure to ionizing radiation and on reducing overexposure risks.^17^

AR can enhance viewing a fetus inside a mother’s womb. Siemens, Karl Storz and IRCAD have developed a system for laparoscopic liver surgery that uses AR to view sub-surface tumors and vessels. AR has been used for cockroach phobia treatment. Patients wearing AR glasses can be reminded to take medications. Surgical guidance can beneﬁt from AR by providing information from medical scan images to a surgeon in a convenient and intuitive manner. A useful review of mixed reality image guided surgery was previously provided and highlighted four key issues of the need to choose appropriate information for the augmented visualization; the use of appropriate visualization processing techniques; addressing the user interface; and evaluation/validation of the system. These remained important areas of research.^18^

AR has been used within neurosurgery area too. A systematic review showed that AR was a versatile tool for minimally invasive procedures for a wide range of neurological diseases and could improve the current generation of neuronavigation systems.^19^ The principles of AR was discussed in the context of laparoscopic surgical oncology before and the beneﬁts and limitations for clinical use were described. Using patient images from medical scanners, AR was shown to improve the visual capabilities of the surgeon and compensate for their otherwise restricted ﬁeld of view. Two main processes are useful in AR including the 3D visualization of the patient data; and registration of the visualization onto the patient.^20^

A study on “Preliminary trial of AR performed on a laparoscopic left hepatectomy” showed that the patient’s operating time was summed up to 205 min, where a blood loss of 300 mL was recorded. The postoperative course was simple. Histopathological analysis revealed the presence of a hepatocellular carcinoma with free margins. Results of intrahepatic visualization suggested that ARGS was beneficial in detecting the tumor, transection plane and medial hepatic vein prior to parenchymal transection, where it did not work due to the substantial changes to the liver’s shape.^21^

In real surgery, it is important to have a thorough, accurate, and detailed knowledge of the anatomical structure of the surgical target. This aspect is especially important in plastic surgery, where most of the surgical outcomes are directly connected to the patient’s external appearance. With the development of computer graphics and sensors, VR and AR have become technologies that can bring new opportunities for development of the diagnostic and operative techniques used in reconstructive, plastic and aesthetic surgery.^21^

The markerless AR-based technology was previously proposed to widely support oral and maxillofacial surgery. It matched a 3D patient teeth model to a real patient’s teeth in a real-time video image to track the patient’s position. It also overaided other 3D anatomical models such as the maxillofacial bone, nerves, and vessels. An AR-based surgical navigation system using a tablet PC with an embedded camera for bone tumor resection in the pelvic region was developed before. It overlaid and provided a 3D patient tumor model and planned resection margin information on the real surgical view. In AR-based display images, it was found to be hard for the user to recognize depth information.^21^

This difficulty was overcome by switching AR display images and providing the closest distance information about a real surgical tool and a 3D patient mesh model. In addition, it was applied to spine surgery. Herein, we highlighted the main advantages of AR in plastic surgery, which are as follows: (1) AR allows the presentation of virtual objects to all human senses in a way identical to their natural counterparts; (2) As a diagnostic tool, the simulated 3D reconstruction of organs based on radiological data can provide a more naturalistic view of a patient’s appearance and anatomy; (3) Preoperative surgical planning can provide a more realistic prediction of the outcome, especially in craniofacial and aesthetic surgery; (4) Computerized 3D atlases of human anatomy, physiology, and pathology can provide better learning and training systems for plastic and reconstructive surgery; and (5) Intraoperative navigation reduces the possibility of major complications and increases the possibility of the best surgical results. Application of AR-based can be displayed in orthognathic surgery and the surgeon’s view of the segmented virtual maxilla in an AR environment.^21^

A search on “navigation surgery using an AR for pancreatectomy” to evaluate the utility of navigation surgery using AR technology. The 3D reconstructed images from CT were created by segmentation. The initial registration was performed by using the optical location sensor. The reconstructed images were superimposed onto the real organs in the monitor display. Of the 19 patients who had undergone hepatobiliary and pancreatic surgery using AR-based NS, the accuracy, visualization ability, and utility of our system were assessed in five cases with pancreatectomy.^22^

Results showed that the position of each organ in the surface-rendering image corresponded almost to that of the actual organ. Reference to the display image allowed for safe dissection, while preserving the adjacent vessels or organs. Then, AR-based NS contributed to accurate and effective surgical resection in pancreatectomy. The AR-based NS system displayed detailed, vivid, and comprehensible anatomical information around the pancreas, which contributed to margin-free and effective surgical resection in pancreatectomy.^22^

A research on “effectiveness of a novel augmented reality-based navigation system in treatment of orbital hypertelorism”, AR technology could superimpose the virtual image generated by computer onto the real operating field to present an integral image to enhance surgical safety to develop a novel AR-based navigation system for craniofacial surgery. It focused on orbital hypertelorism correction, because the surgery required high preciseness and was considered tough even for senior craniofacial surgeon.^23^

Twelve patients with orbital hypertelorism were selected. The preoperative computed tomography data were imported into 3-dimensional platform for preoperational design. The position and orientation of virtual information and real world were adjusted by image registration process. The AR toolkits were used to realize the integral image. Afterward, computed tomography was also performed after operation for comparing the difference between preoperational plan and actual operational outcome. Results showed that the AR-based navigation system was successfully used in these patients, directly displaying 3-dimensional navigational information onto the surgical field. A better appearance by the guidance of navigation image was achieved. This study reported an effective visualized approach for guiding orbital hypertelorism correction. The AR-based navigation system might lay a foundation for craniofacial surgery navigation. The AR technology could be considered as a helpful tool for precise osteotomy in craniofacial surgery.^23^

AS in perforator flap transplantation, dissection of the perforator is an important, but difficult procedure because of the high variability in vascular anatomy. A research done on “A novel AR-based navigation system in perforator flap transplantation- a feasibility study”, preoperative imaging techniques could provide substantial information about vascular anatomy; however, it did not provide direct guidance for surgeons during the operation. In this study, a navigation system (NS) was established to overlie a vascular map on surgical sites to further provide a direct guide for perforator flap transplantation^24^

Using this method, the NS was established based on computed tomographic angiography and AR techniques. A virtual vascular map was reconstructed according to computed tomographic angiography data and projected onto real patient images using AR Tool Kit software. Additionally, a screw-fixation marker holder was created to facilitate registration. With the use of a tracking and display system, the NS was conducted on an animal model and the system error was measured on a rapid prototyping model. Results showed that the NS assistance allowed for correct identification, as well as a safe and precise dissection of the perforator. AR-based NS could provide precise navigation information by directly displaying a 3-dimensional individual anatomical virtual model onto the operative field in real time. It allowed rapid identification and safe dissection of a perforator in free flap transplantation surgery.^24^

A study on “Google Glass interface for sensory feedback in myoelectric hand prostheses” to provide sensory feedback to the user of the prosthesis is an important challenge. The common approach is to use tactile stimulation, which is easy to implement, but requires training and has limited information bandwidth. AN alternative approach based on AR has been suggested. The GLIMPSE, a Google Glass application was developed, which was connected to the prosthesis via a Bluetooth interface and rendered the prosthesis states (EMG signals, aperture, force and contact) using AR (see-through display) and sound (bone conduction transducer).^25^

The interface was tested in healthy subjects that used the prosthesis with (FB group) and without (NFB group) feedback during a modified clothespins test that allowed us to vary the difficulty of the task. The outcome measures were the number of unsuccessful trials, the time to accomplish the task, and the subjective ratings of the relevance of the feedback. The results showed that there was no difference in performance between FB and NFB groups in the case of a simple task (basic, same-color clothespins test). The GLIMPSE feedback did not increase the time to accomplish the task.^25^

Therefore, the supplemental feedback might be useful in the tasks which are more demanding, and thereby, less likely to benefit from learning and feedforward control. The subjects integrated the supplemental feedback with the intrinsic sources (vision and muscle proprioception), developing their own idiosyncratic strategies to accomplish the task. This study demonstrated a novel self-contained, ready-to-deploy, wearable feedback interface. The interface was successfully tested and was proven to be feasible and functionally beneficial. The GLIMPSE could be used as a practical solution but also as a general and flexible instrument to investigate closed-loop prosthesis control.^25^

A study on “Precise positioning of an intraoral distractor using augmented reality in patients with hemifacial microsomia” has been done on 20 patients with hemifacial microsomia who were randomly assigned to experimental and control groups. Pre-operative computed tomography was used in both groups, whereas AR was used in the experimental group. Afterwards, pre- and post-operative computed tomographic scans of both groups were superimposed, and several measurements were made and analyzed. Findings showed that both the conventional method and AR technique achieved proper positioning of the osteotomy planes, although the AR was more accurate. This study reported an efficient approach for guiding intraoperative distraction osteogenesis. AR tools such as the AR Toolkit may be helpful for precise positioning of intraoral distractors in patients with hemifacial microsomia in craniofacial surgery.^26^

Despite the growing interest in AR and the large body of advances and research, several challenges and issue still exist and need to be addressed. Having all those gains and benefits does not come without concerns. Using this technology one may not be able to ignore some of the threats of the real, surrounding environment. AR will have an important role in image-based augmentation of the surgical environment. This will require increasingly powerful microcomputers to drive AR, which is currently limited but may improve with time.^27^

As with electronic patient records, confidentiality and data management will be a major hurdle in the integration of recordable HMDs into medical practice. All should consider ethical and legal obligations, when using technology to either review, store or transfer patient data to prevent medical identity theft. Encryption improves but does not guarantee prevention from data hijacking. It is important that whichever system is developed meets the standards for health care information governance. With so many different healthcare systems, it is likely that there will be many different AR systems in use, which will have varying degrees of compatibility. As such, the healthcare market will capitalize on developing accessible price sensitive software and hardware to market. It is anticipated the global AR healthcare industry will be worth $641 million by 2018.^27^

Here, we classify the limits that characterize the current state of the art of AR based on aspects of technology, social acceptance, and usability. (i) AR system has to deal with vast amount of information in reality. So the hardware used should be small, light, and easily portable and fast enough to display graphics; (ii) The battery life used by these complicated AR devices is another limitation for AR’s uses; (iii) AR tracking needs some system hardware such as GPS to provide accurate markers, ask them to be both accurate and reliable enough; (iv) AR systems usually obtain a lot of information, and need software to filter the information, retain useful information, discard useless data and display it in a convenient way; and (v) Despite the numerous benefits of AR in the medical field, some issues have arisen, including, for example, incorrect visualization of interposition between real and virtual objects.^28^

A challenge in surgery is that the position of organs and tissues cannot be estimated but the surgeon must know them exactly. AR projections do not always correspond the reality because of the structure of tissues in human body and patient’s subtle movements, such as aspiration and other tissue function. An attempt to provide more realistic real-time data was made before by integrating laparoscopy and 3D-ultrasound images, i.e. combining images inside and outside a body, provided by two different imaging methods.^28^ Table 1 summarizes serious concerns about those technologies, some can be physical threats, other behavioral, privacy, security and some can be placed even at a level of “National security threat”.^29^

**Table 1:** Serious concerns about physical threats, and behavioral, privacy, security at a level of “National security threat”

**Type of Threat**	**Example**	**Effect**	**Level of threat**
Physical threats	Devices (like head mounted displays) and sensors do not respond quickly or accurately in the simulated environment	On the health or even life of humans, immediately or in later situations in real environment	High
Security threats	Criminals or terrorist acquiring those technologies and getting hold of software or communication	Exposure of the security holes of important sectors (police, military, industrial, communication)	High
behavioral threats	Using avatars (instead of real person) for destructive behavior, harassing and stalking	Annoying, bullying and stressing others	Medium
Educational threats	Acquiring improper skills and knowledge of environment	Trainees can not cope in real-life situations	Medium to high
Bad investment	Developing augmented reality platform can be very expensive	Projects on augmented/virtual reality can bankrupt major developers	Medium

The physical threats may come from the imperfection of devices like head-up displays, or their non-interference with the peripheral vision of the pilot/users as the displays present information only in the central field. Also there is the threat of misjudging relative motions, due to the poorer (or absent) peripheral field. In regard to the security threats, there are significant risks in the way augmented environments are deployed. The communications used in such platform, voice, position, messages have to be protected and encrypted.^30^

AR is often used in training. There is also the issue that those technologies help reducing social and cultural barriers, giving the chance of some to participate at higher level in the educational process and often this is done using avatar. Regarding the behavioral threats, some individuals can assert a more destructive behavior, and show a non-proper conduct of behavior. Privacy threats are of concern even at present virtual societies like Facebook and Google+. Monitoring of presence, behavior and other issues of their users is alarming and stressful. While those technologies can bring a great benefit, jumping on their use and development might be costly for a number of companies. It might be better to start with pilot virtual/AR projects than spending a lot of money and not getting the expected advantage and value. Internet of things (IoT) has also a big security problem, sensors and other devices used in it are built with little or no security requirement. This could lead to serious vulnerabilities in applications at home, in cities, industry, etc.^30^

Proper and adequate securing of AR is inevitably producing a significant challenge. Achieving a suitable reaction in this sense is a quite challenging task. Taking onto account the new understandings for “privacy space” and hybrid phenomena like “Advanced Persistent Threats” looks rather ambiguous and not quite certain. This directly reflects to a fast evolving cyber threats landscape for the human factor and environment of living.^31^

A methodological approach for proactive exploration of the cyber threat landscape in the evolving digital environment is suggested. It should be noted here that the presented approach has been successfully developed and tested during the last five years in different cyber fields, ranging from social networks to smart environments implementing multiple IoT gadgets (Gaget is a beautiful application for checking Google Analytics data on your iPhone and on OS X.). The key ideas behind this model are accomplishing system modelling (based on expert knowledge), further analyzed in the discrete case and simulated in mixed reality environment.^32^

The obtained results are validated with human biometrics monitoring, probabilistic assessment and questionnaire based multicriteria evaluation. Finally, the validated results are also verified in different digital reality projections (encompassing multiple scenarios, concerning real, virtual and mixed experimental worlds) and are expected to be practically applied with overall global cyber trends landscape analysis, achieving proactive understanding of future cyber threats evolution.^32^

As cyber threat context definition is difficult to be outlined, regarding its future evolution, the upcoming threats landscape in the cyber space (up to year 2020) will be strongly influenced by transformed privacy, biometric disturbances, and espionage, regarding the complete studied technological set (‘IoT Gadgets’, ‘Mixed Realities’, ‘Advanced Communications’, ‘Enhanced Multimedia’). Whilst, social engineering and advanced malware are quite uncertain. Data breaches are expected to be weakened as threat, being already a quite exploited one.^32^

Practical support in this sense was achieved via monitoring of user multiple biometrics, Balanced Score Card post-simulation assessment and probabilistic machine simulation of cyber-attacks. Below these three approaches will be given with more details, regarding some successful implementation examples. 

Suitable results verification requires multiple scenarios and environments implementations and production comparison. Practical support in this sense was achieved via monitoring of user multiple biometrics, Balanced Score Card post-simulation assessment and probabilistic machine simulation of cyber-attacks.^32^

Both psychological and physiological assessment of user complex characteristics, like emotions and behavior were organized for reliable hybrid simulation evaluation within different situational scenarios. Personality assessments of temperament, depression and sensation seeking evaluation of motivation have been also applied. Additional stress assessment has been studied through monitoring trainees’ response times. This is in close relation with human neural dynamics observations of different training process aspects in digital space.^32^

The approach is extending the Balanced Score Card (BSC) tool with questionnaire-based assessment and Delphi filtering (A program for setting filters on tables and query result sets), similar to other security exercises assessment. During this process practical organization an important note is the participant motivation for proper questionnaires treating. Unfortunately, it is difficult to be directly measured and checked, together with correct question understanding. This naturally generates noisy data results from the user response monitoring perspective. A useful support in this sense could be the combination with indirect user feedback, based on stored activities biometrics analysis or other bench mark machine simulation results.^32^

We can conclude that despite of few resources on AR, especially in Iran, vast dealing with AR capabilities has been done in the realm of surgery as one important branches of medicine, while showing its benefits and shortcomings like different physical, educational and behavioral threats. Since “privacy and security matters” are the most concerning issues in this regard, then some solutions such as Results “Validation”, “Verification”, “Balanced Score Card Assessment” and “Biometrics Support” have been prescribed. In future studies, it is recommended to deal with various fields of Medicine affected by AR and propose some cures for other kinds of AR concerns such as “cost” and “physical protocols”. 

## CONFLICT OF INTEREST

The authors declare no conflict of interest.
